# Detection and Quantitation of Circulating Tumor Cell Dynamics by Bioluminescence Imaging in an Orthotopic Mammary Carcinoma Model

**DOI:** 10.1371/journal.pone.0105079

**Published:** 2014-09-04

**Authors:** Laura Sarah Sasportas, Sharon Seiko Hori, Guillem Pratx, Sanjiv Sam Gambhir

**Affiliations:** 1 Department of Radiology, Molecular Imaging Program at Stanford, Stanford University School of Medicine, Stanford, California, United States of America; 2 Department of Bioengineering, Stanford University, Stanford, California, United States of America; 3 Department of Radiation Oncology, Stanford University, Stanford, California, United States of America; Sapporo Medical University, Japan

## Abstract

Circulating tumor cells (CTCs) have been detected in the bloodstream of both early-stage and advanced cancer patients. However, very little is know about the dynamics of CTCs during cancer progression and the clinical relevance of longitudinal CTC enumeration. To address this, we developed a simple bioluminescence imaging assay to detect CTCs in mouse models of metastasis. In a 4T1 orthotopic metastatic mammary carcinoma mouse model, we demonstrated that this quantitative method offers sensitivity down to 2 CTCs in 0.1–1mL blood samples and high specificity for CTCs originating from the primary tumor, independently of their epithelial status. In this model, we simultaneously monitored blood CTC dynamics, primary tumor growth, and lung metastasis progression over the course of 24 days. Early in tumor development, we observed low numbers of CTCs in blood samples (10–15 cells/100 µL) and demonstrated that CTC dynamics correlate with viable primary tumor growth. To our knowledge, these data represent the first reported use of bioluminescence imaging to detect CTCs and quantify their dynamics in any cancer mouse model. This new assay is opening the door to the study of CTC dynamics in a variety of animal models. These studies may inform clinical decision on the appropriate timing of blood sampling and value of longitudinal CTC enumeration in cancer patients.

## Introduction

The invasion of circulating tumor cells (CTCs) into blood represents a critical step in the process of cancer metastasis, which is responsible for 90% of cancer deaths. CTCs can be detected and harvested from patient blood and are ideal candidates for a real-time “liquid biopsy” of the tumor [1]. It has been demonstrated that the presence of CTCs is significantly associated with poorer prognosis, both in early-stage and metastatic breast cancer [2].

Many exciting technologies have been developed in the past decade to detect CTCs in patient blood samples [3]. These techniques can enrich and detect CTCs in human blood based on their physical properties, such as filtering them by size, and/or their biological properties, such as protein expression, using immunocytochemistry or multi-marker RT-PCR [1], [3]. CTCs can be detected and captured on various platforms, including microfluidic chips, immunomagnetic beads, immunomagnetic columns and membrane filter devices [1]. These CTC platforms may enable a broad range of novel clinical applications, from the early detection of metastatic disease to the prediction of therapeutic response.

Despite increased development of novel CTC detection methods over the past few decades, the dynamics of CTCs, defined as the temporal variations of CTC numbers during tumor growth and progression remain largely uncharacterized. Tumor cell infiltration into blood vessels was long believed to be a relatively late step in tumor development [4], but recent studies have shown that such invasion can happen at early stage [5]–[7], with CTCs being detectable in the bloodstream of both early-stage and advanced cancer patients [8], [9]. There is a need for a detailed characterization of the appearance and dynamics of CTCs during the course of tumor development. CTC dynamics are difficult to study in patients where each individual tumor harbors different characteristics and where blood draws are typically aligned with therapeutic interventions. Mouse models have the potential to shed light on the role and dynamics of circulating tumor cells during cancer progression [10], since they allow for a much easier control of tumor progression, homogeneity in the subject cohort and blood sampling and imaging time points. CTCs have been probed in mouse models of metastasis using various methods [11]–[29] but none of those assays allow both identification of live CTCs independently of their epithelial status with minimal background from blood cells, and potential recovery of viable CTCs in a longitudinal study. Bioluminescence imaging (BLI), a molecular imaging method based on the transfection of cells with a luciferase enzyme, offers both an exquisite sensitivity, down to 1 single cell detected *in* *vivo* [30]–[33] and quantitative capability [31], [33]. We reasoned that bioluminescence imaging could be used to detect and quantify rare CTCs in blood samples in mouse models of metastasis.

In this study, for the first time, we used bioluminescence imaging to assess CTCs in blood samples from an orthotopic mouse metastatic mammary carcinoma model. We demonstrated that this novel application of BLI combines several highly desired properties for a CTC assay: (1) quantitative capabilities, (2) exquisite sensitivity down to 2 CTCs in 0.1–1 mL blood samples, (3) high specificity with minimal background from blood cells, (4) identification of live CTCs independently of their epithelial status, and (5) potential recovery of viable CTCs. Using this method we demonstrated that we were able to uncover the dynamics of CTCs during the progression of cancer from primary tumor to metastasis in a mouse model of breast cancer.

## Materials and Methods

### Cell lines

We previously transfected murine metastatic carcinoma cell line 4T1 with a lentiviral construct containing a bifusion reporter of enhanced green fluorescent protein (eGFP) and firefly luciferase-2 [34]. Using two rounds of fluorescence activated cell sorting (FACS), we established a stable cell line (denoted 4T1-GL) that expresses high levels of the bifusion reporter gene for at least 12 passages in culture [34]. Using an additional round of FACS, we selected the 4T1-GL-TS population as the 5% brightest cells among 4T1-GL cells, based on GFP fluorescence.

We also selected and expanded 48 different clonal populations from the 4T1-GL-TS cells. Among the clones harboring the brightest BLI signal and sustained growth in culture (A4, C3, D3, F3), we selected clone 4T1-F3 as the brightest clone.

### Cell culture

4T1, 4T1-GL and derived mixed and monoclonal populations including 4T1-F3 metastatic mouse breast cancer cells were cultured in Dulbecco’s modified Eagle medium High-Glucose (DMEM, Gibco) supplemented with 10% fetal bovine serum (FBS, Gibco), 100 units/mL of penicillin, 100 µg/mL of streptomycin, and 0.25 µg/mL amphotericin B (Antibiotic-Antimycotic, Gibco).

### Murine metastasis model

Female Nu/nu mice were purchased from Charles River Laboratories (Wilmington, Massachusetts). All animal studies were approved by The Stanford University Institutional Animal Care and Use Committee. 2×10^7^ 4T1-GL or 5×10^6^ 4T1-F3 metastatic breast cancer cells were freshly harvested and resuspended in a 100 µL solution containing 50% culture medium and 50% Matrigel (BD Biosciences). The suspension was then orthotopically implanted by injection into the 4^th^ left mammary fat pad.

### Blood collection

100-µL whole blood samples were collected by submandibular bleeding into K2-EDTA-coated tubes via a blood collection funnel (BD Biosciences, Vacutainer). Terminal blood puncture to obtain 1 mL whole blood was performed by cardiac puncture and collected into K2-EDTA-coated tubes. To perform cardiac puncture, mice were deeply anesthetized under isofluorane and a 21-gauge needle coated with heparin was inserted into the heart. Mice were euthanized Immediately following the cardiac puncture. Red blood cells were lysed by addition of an Ammonium Chloride solution (ACK, Stem Cell Technologies) following the manufacturer’s protocol.

### Bioluminescence microscopy

Terminal blood samples (1 mL) were processed by red blood cell lysis and then incubated overnight on a proprietary cell adhesion matrix (CAM)-coated plate (Vita-Assay TM AN6W, Vitatex, NY) in their culture medium. Following harvesting by CAM-dissociation using a proprietary CAM enzyme (Vitatex, NY), the cell suspension was pipetted into a microscopy dish (#0 cover glass, 0.085–0.115 mm, In Vitro Scientific). The imaging dish was placed in a bioluminescence microscope (LV200, Olympus) outfitted with a 100X/1.35 NA oil objective (UPLAPO00XOI3, Olympus) and a deep-cooled electron-multiplying charge-coupled device (EM-CCD; ImageEM C9100-14, Hamamatsu). The LV200 is also equipped with temperature, humidity, and CO_2_ regulation for extended live cell imaging.

Brightfield and bioluminescent images were acquired using the 100X objective and 5-min exposure time for bioluminescent image acquisition.

### Cancer cell spiking experiments

The average cell concentration of 4T1-GL or 4T1-F3 was measured using a benchtop cell counter (Cellometer Auto T4 cell counter, Nexcelom Bioscience, MA). For high number of cells (>150 cells), a 1∶10 serial dilution of various numbers of 4T1-GL (8×10^1^–8×10^5^) was spiked into either a volume of 100 µL of pre-warmed culture medium or 100 µL of whole blood freshly harvested from a healthy mouse (n = 4). For low number of cells (0–150 cells), in order to achieve such precision in counting the number of cancer cells spiked into blood samples, we modified our spiking technique from the previous method. Instead of measuring average cell concentration using a benchtop cell counter, we labeled live 4T1-GL or 4T1-F3 cells with non-toxic doses of the very bright fluorescent dye carboxyfluorescein (10 µM, Vybrant CFDA SE Cell Tracer Kit, Invitrogen) in order to manually count the exact number of cells spiked in the sample using a benchtop fluorescence microscope (Fig. S1). Because of the method we used to count the cells, each spiking experiment was unique.

### Bioluminescence imaging of ex vivo blood samples

After addition of 100 µL D-luciferin substrate, the BLI signal from the wells was acquired in the IVIS Spectrum (Xenogen, Alameda, CA) using a highly sensitive, cooled CCD camera, and compared to the baseline signal from culture medium or whole blood or RBC lysed blood. The protein content was measured using a Pierce total protein assay (Thermo Scientific) and compared to a protein standard curve.

### In vivo Bioluminescence Imaging

For bioluminescence imaging, 100 µl of 30 mg/ml D-Luciferin (Biosynth AG, Switzerland) was injected intraperitoneally and mice were anesthetized with 1–2% inhaled isoflurane in oxygen. Bioluminescence signal was measured using the IVIS system 200 series (Xenogen, Alameda, CA, USA). Living Image software (Xenogen, Alameda, CA, USA) was used to compute regions of interest (ROI) and integrate the total bioluminescence signal in each ROI. Data were analyzed using average radiance (photons/second/cm^2^/steradian) in the ROIs and normalized to background signal. For biodistribution studies, organs were harvested and immediately soaked in a 3 mg/mL solution of D-Luciferin for 5 minutes prior to BLI imaging. Biodistribution data were analyzed using total flux (photons/second) in the ROIs drawn around each organ and normalized to background signal.

### Statistical analysis

Results were expressed as mean ± standard deviation (SD) or mean ± standard error of the mean (SEM) as indicated in the figure legends. An unpaired, 2-tailed Student’s t test was used to calculate P values.

## Results

### Detection and quantification of 4T1-GL CTCs in blood samples by highly sensitive Bioluminescence Imaging (BLI)

**Figure 1:**
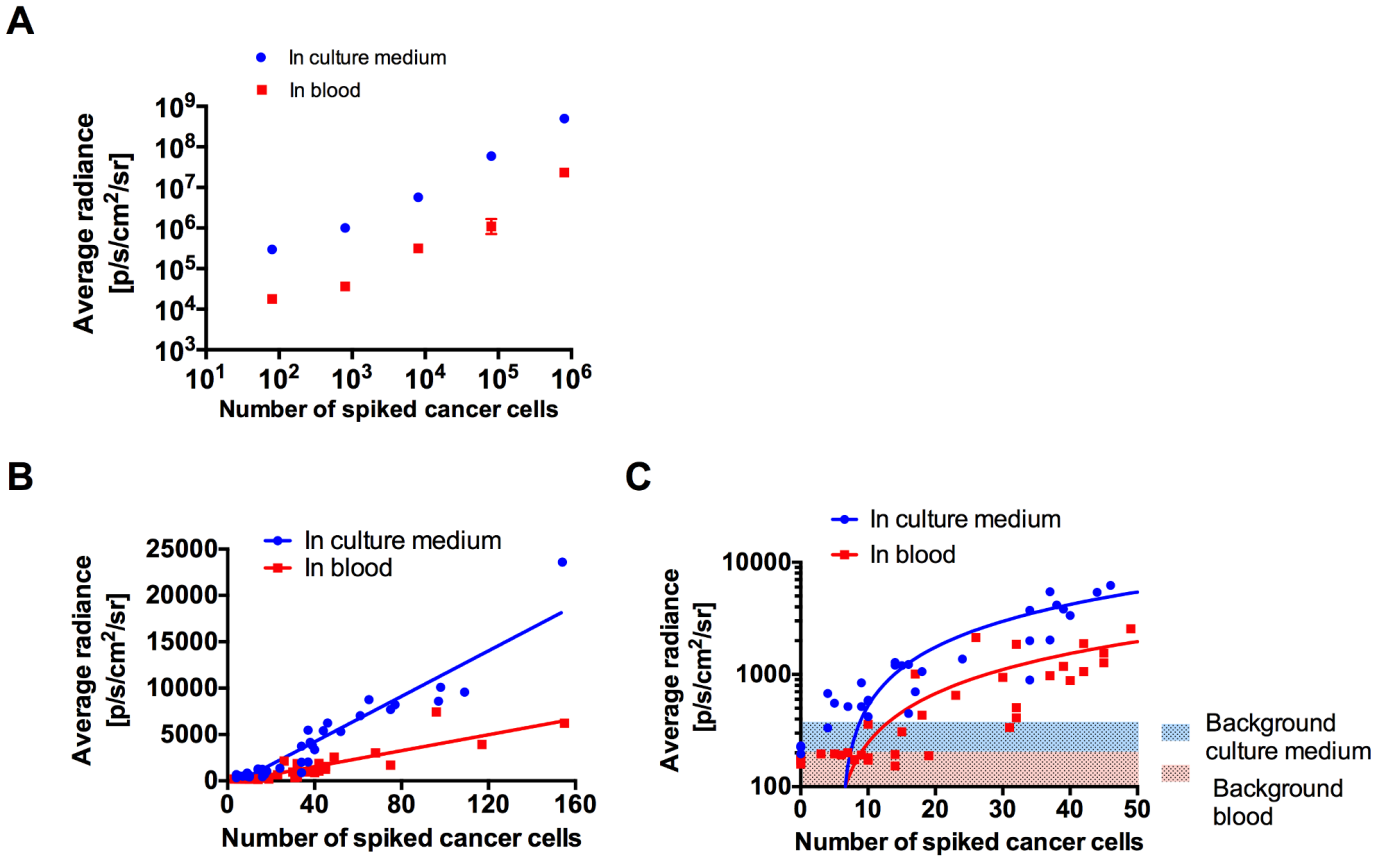
Bioluminescence imaging detection and quantification of 4T1-GL cancer cells spiked in mouse whole blood samples. (**A**) Quantification of BLI imaging of high numbers of 4T1-GL cells spiked into 100 µL culture medium or 100 µL mouse whole blood samples, shown as mean ± SEM on a log-log scale. There was a significant linear correlation (R^2^ = 0.95 for culture medium; R^2^ = 0.92 for whole blood). (**B**) Quantification of BLI imaging of low numbers (0–150) of 4T1-GL cells cells spiked into 100 µL culture medium or 100 µL mouse whole blood samples. There was a significant linear correlation (R^2^ = 0.90 for culture medium; R^2^ = 0.92 for whole blood). (**C**) Zoom on very low number of cells (0–50) in (**B**). The shaded regions represent background BLI radiance.

We previously established a stable 4T1-GL cell line expressing high levels of the bifusion reporter gene eGFP and Luc2 [34]. To determine the lowest number of 4T1-GL cells that can be detected in a blood sample using BLI, we spiked 8–8×10^5^ 4T1-GL cancer cells in l00 µL of pre-warmed culture medium or mouse blood (*n* = 4). BLI images of the spiked samples were acquired and their signal was compared to the control signal from culture medium or whole blood alone (Fig. 1A). The BLI average radiance highly correlated with cell number for 4T1-GL cells spiked in culture medium (R^2^ = 0.95) or whole mouse blood (R^2^ = 0.92). The average radiance from as few as 8 cells was significantly higher than that from culture medium (p<0.0001) or whole mouse blood (p = 0.025). This demonstrates that, in the spiked cell number range evaluated, highly sensitive BLI is an accurate method to detect and quantify CTCs in *ex vivo* blood samples.

Because CTCs are very rare events (with as few as 1 CTCs detected in 10 ml blood [35]) we next sought to evaluate the sensitivity of our BLI detection technique for as few as 0–150 cells per blood sample (Fig. 1B). Average radiance linearly correlated with cell number for cancer cells spiked in culture medium (R^2^ = 0.90) as well as for cancer cells spiked in whole mouse blood (R^2^ = 0.78), For very low numbers of spiked cancer cells (0–50 cells, Fig. 1C), our technique was capable of detecting the presence of at least 10 cancer cells in culture medium and in whole blood samples. Indeed, the average radiance for the wells corresponding to 11–50 cells was significantly higher than the background of culture medium (p = 0.02) or whole blood (p = 0.03). The lower limits of reliable detection (i.e. no false negatives) in an individual mouse blood sample was found to be 19 CTCs. Indeed, there was a sample with 19 spiked cancer cells that did not give a signal above blood background (Fig. 1C). These experiments demonstrate that highly sensitive BLI is capable of detecting as few as 10 4T1-GL cells in 100 µl of mouse blood *ex vivo*.

### BLI for simultaneous monitoring of primary tumor growth, CTC dynamics and metastatic spread in the 4T1-GL orthotopic mouse model of breast cancer metastasis

**Figure 2:**
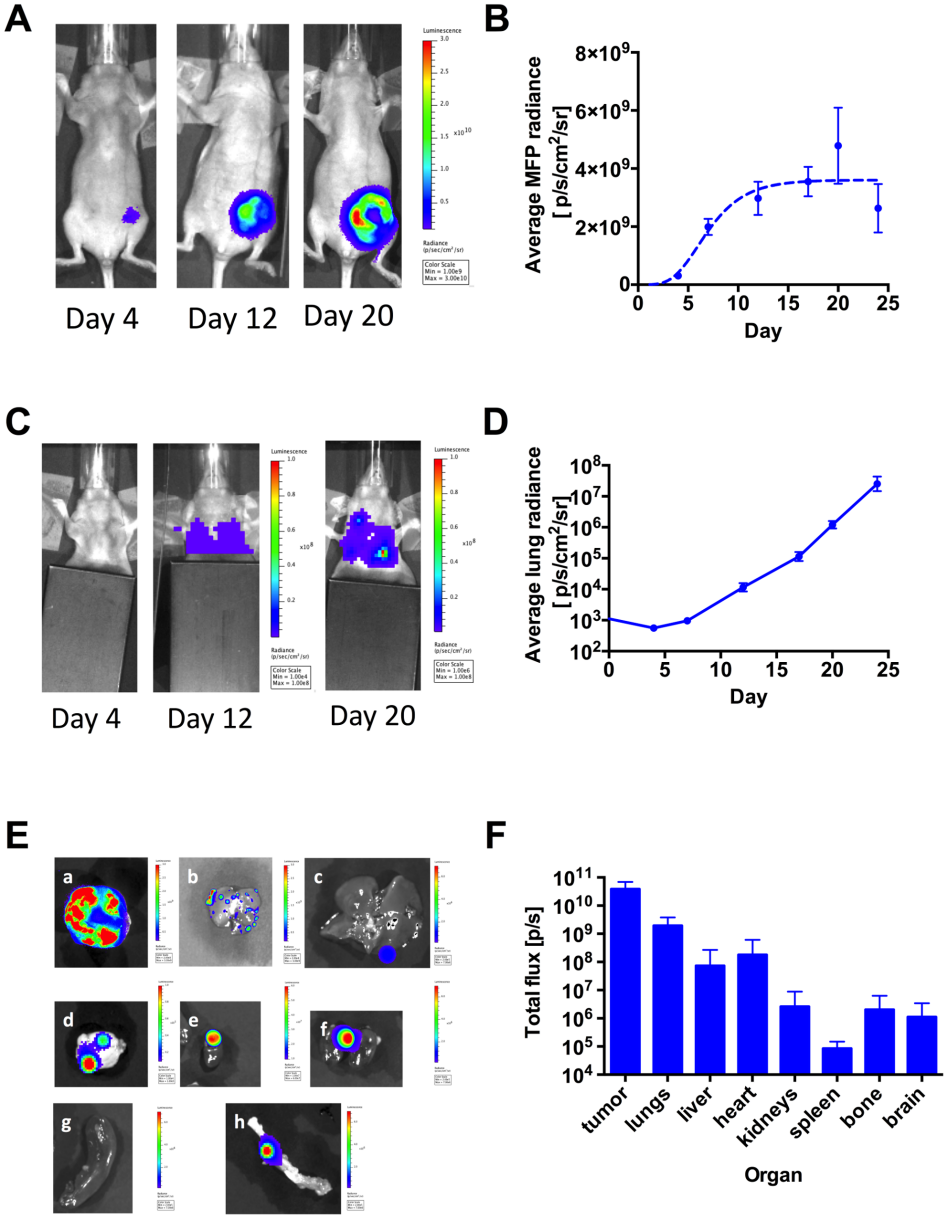
Bioluminescence imaging of primary tumor and metastases in the 4T1-GL orthotopic mammary tumor model. (**A**) Primary tumor growth in the mammary fat pad (m.f.p) as monitored using BLI, following implantation of 2×10^7^ 4T1-GL cells in the fourth left mammary fat pad (image scales in p/s/cm^2^/sr, n = 7 mice). (**B**) Corresponding quantification of BLI signal in the m.f.p area, shown as mean ± SEM and fit of the mean values to a Gompertzian tumor growth equation (dashed line, R^2^ = 0.86). (**C**) Metastases in the upper body (lung area) as monitored using BLI in the same animals (image scales in p/s/cm^2^/sr). (**D**) Corresponding quantification of the signal in the lung area, shown as mean ± SEM. (**E**) Metastases in excised organs on day 23, as monitored using BLI in the same animals: a. tumor (scale: 2×10^8^–3×10^9^ p/s/cm^2^/sr), b. lungs (scale: 2×10^8^–3×10^9^ p/s/cm^2^/sr), c. liver (scale: 2×10^5^–7×10^6^ p/s/cm^2^/sr), d. brain (scale: 1×10^4^–1×10^5^ p/s/cm^2^/sr), e. heart (scale: 1×10^7^–6×10^7^ p/s/cm^2^/sr), f. kidneys (scale: 2×10^5^–7×10^6^ p/s/cm^2^/sr), g. spleen (scale: 2×10^5^–7×10^6^ p/s/cm^2^/sr), h. bone (scale: 2×10^5^–7×10^6^ p/s/cm^2^/sr). (**F**) Corresponding quantification of BLI signal in these organs, shown as mean ± SD.

We next sought to use BLI to simultaneously image primary tumor growth, CTC dynamics, and metastatic spread in an orthotopic model of 4T1-GL breast cancer. We implanted 2×10^7^ 4T1-GL cells in the murine mammary fat pad (m.f.p) and monitored breast tumor growth by whole-body BLI every 3–5 days over a period of 24 days (Fig. 2A–B). We also monitored the torso of the animal for lung metastases that were observed as early as 12 days following tumor implantation (Fig. 2C–D). Subsequently, the lung metastatic burden increased exponentially over 12 days for all mice (day 12-day 24, Fig. 2D). *Ex vivo* BLI of excised organs at day 24 showed that this model not only gives rise to metastases in the lungs but also in other organs, including bone, liver and brain (Fig. 2E–F).

**Figure 3:**
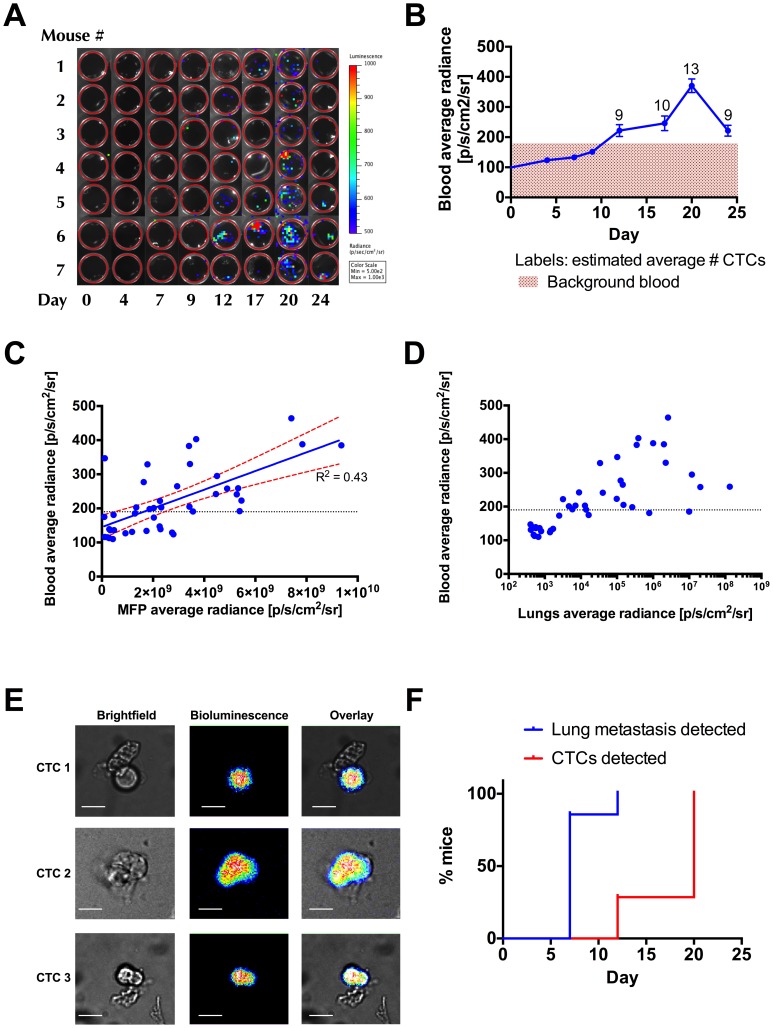
Bioluminescence imaging of CTCs dynamics in the 4T1-GL orthotopic mammary tumor model. (**A**) CTCs in 100 µL blood samples from n = 7 mice, as monitored using BLI (image scale in p/s/cm^2^/sr). (**B**) Corresponding quantification of BLI signal in (A), shown as mean ± SEM. The pink shaded region corresponds to average background signal. (**C**) Correlation of blood BLI signal and primary tumor signal in the MFP area for all timepoints measured and all animals studied. The line indicates a linear regression (R^2^ = 0.43) and the red dotted line shows the 95% confidence interval. The black dotted line represents the average BLI background signal of whole blood from healthy mice. (**D**) Correlation of CTC detection BLI signal and lung metastasis signal in the torso area for all timepoints measured and all animals studied. There was no significant linear correlation in this case. The black dotted line represents the average BLI background signal of whole blood from healthy mice. (**E**) CTCs and CTC microemboli in terminal blood samples from animals bearing day 23 tumors, as imaged by bioluminescence microscopy. (**F**) Statistical analysis showing the onset of lung metastasis and of CTCs detected in blood over time in the n = 7 animals.

We applied the CTC BLI detection method described above to follow the dynamics of CTCs in 7 mice over 24 days post-implantation of tumor. In whole blood samples harvested freshly every 3–4 days (Fig. 3A), we observed an onset of CTCs starting at day 12 following m.f.p. tumor implantation and a peak of CTCs at day 20. The number of CTCs detected ranged from 10–15 CTCs per 100 uL blood sampled, with an average of 13 CTCs at the peak on day 20 (Fig. 3B). On day 24, the blood BLI signal decreased but remained above the background level. Interestingly, although not all mice (n = 6) in the cohort showed a positive BLI signal on day 12, all mice (n = 7) showed a constant increase in the average radiance from day 9 to day 20, with a peak of blood BLI signal on day 20, and a clear decrease in the signal measured on day 24 (Fig. S2). The appearance of a positive blood BLI signal on day 12 (21% signal above background) coincided with the appearance of lung metastasis (Fig. 2C, Fig. 3A). Our imaging results demonstrate that highly sensitive BLI can be used to detect and track CTC dynamics over the timecourse of metastatic tumor development. We also demonstrate that CTC number correlates linearly with primary tumor progression (R^2^ = 0.43, Fig. 3C) but not with lung metastatic progression (Fig. 3D).

To confirm the presence of CTCs in our BLI-positive blood samples, we used BLI microscopy [36] to scan 1-ml terminal blood samples collected from tumor-bearing mice (n = 2) sacrificed on day 23. We identified single CTCs as well as clusters of 2–3 CTCs expressing luciferase in these blood samples (Fig. 3E). These results confirmed that the positive BLI signal detected in the whole blood samples we collected is originating from single cells or clusters of CTCs expressing luciferase, demonstrating the specificity of our BLI assay. BLI signal can only be detected from live cells, so these results show that live CTCs can be identified in blood samples. These detected CTCs could potentially be cultured and expanded for further characterization. [37], [38].

### Red blood cell lysis enhances BLI detection sensitivity for low numbers of CTCs

**Figure 4:**
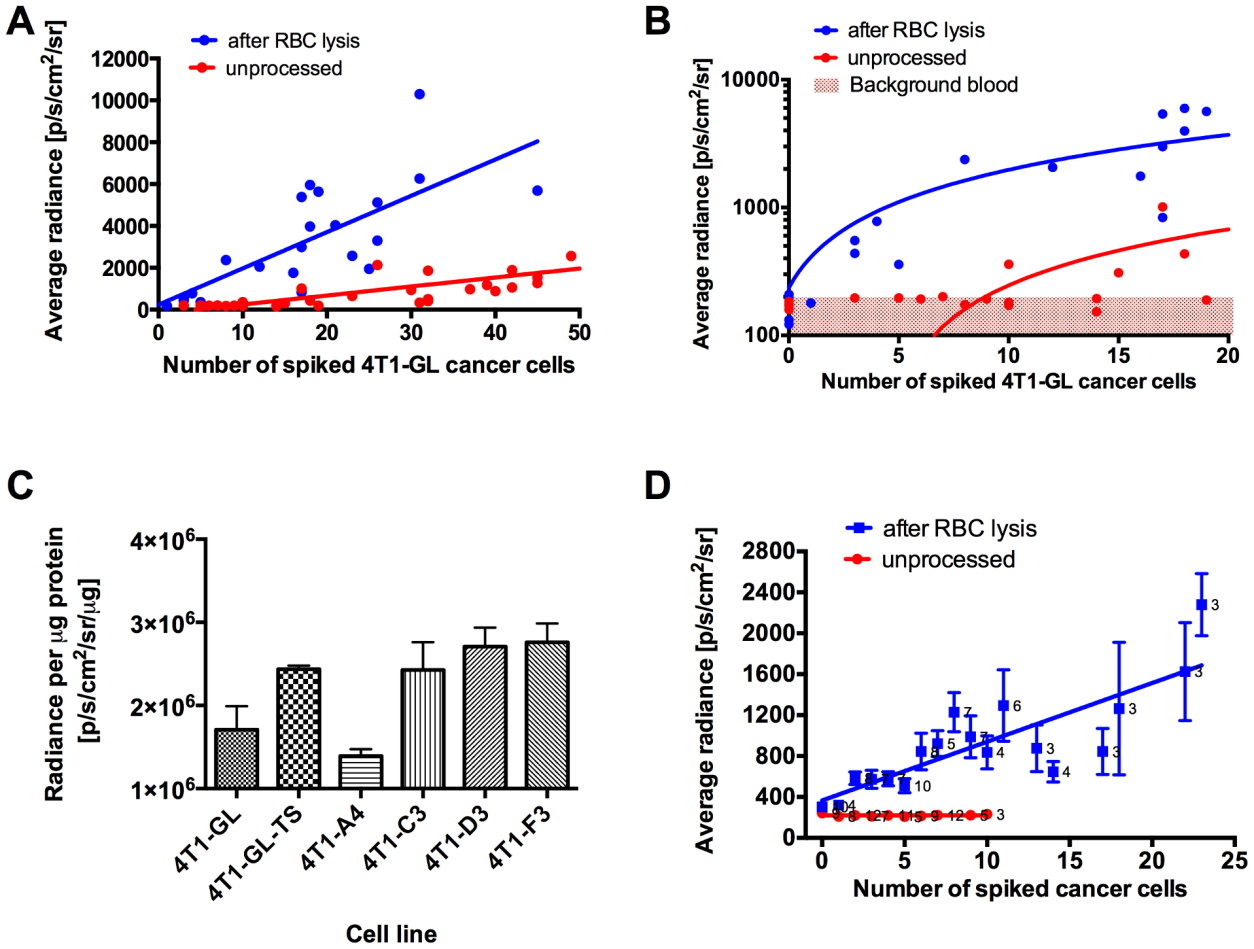
Optimization of BLI detection and quantification of 4T1-GL and 4T1-F3 cancer cells spiked in mouse blood samples. (**A**) Quantification of BLI imaging of low numbers (0–50) of 4T1-GL cells spiked into 100 µL blood with or without RBC lysis There was a significant linear correlation (R^2^ = 0.64 for RBC lysis; R^2^ = 0.78 for unprocessed). (**B**) Zoom on very low number of cells (0–50) in (**A**). The shaded regions represent background BLI photon counts. (**C**) Quantification of BLI signal per µg protein for the following cell lines: 4T1-GL, 4T1-GL-TS, monoclonal populations sorted from 4T1-GL-TS: 4T1-A4, 4T1-C3, 4T1-D3 and 4T1-F3, shown as mean ± SD (**D**) Quantification of BLI imaging of low numbers (0–25) of 4T1-F3 cells spiked into 100 µL blood with or without RBC lysis, shown as mean ± SEM. The label numbers for each point indicate number of replicates (n = 3–12). There was a significant linear correlation (R^2^ = 0.68) for RBC-lysed samples.

In the 4T1-GL orthotopic model, we successfully monitored CTC dynamics using our BLI assay, but observed that the onset of CTCs in blood samples coincided with the onset of metastasis at day 12 (Fig. 3F). To determine whether this observation reflects a true absence of CTCs before this time point, or if fewer CTCs (<11 cells) were missed due to the BLI signal absorbance by hemoglobin-containing red blood cells (RBCs) [39], we sought to improve the sensitivity of our BLI-based CTC detection method by testing whether an additional RBC lysis step could potentially allow detection of fewer cancer cells spiked into blood samples. To do this, we spiked live 4T1-GL cells into 100 µL whole mouse blood samples, lysed the red blood cells with an ammonium chloride solution, and imaged the samples by BLI as previously described. For low cancer cell numbers (0–50 cells), the blood average radiance linearly correlated with spiked cell number for both unprocessed (R^2^ = 0.78, Fig. 4A) and lysed (R^2^ = 0.64, Fig. 4A) blood samples. Background-corrected relative BLI signal also correlated with cell number (R^2^ = 0.76 and R^2^ = 0.67 respectively, Fig. S3A). RBC lysis increased the slope of this linear relationship by a factor of 3.77 (Fig. 4A). Furthermore, for very low numbers of spiked cancer cells (0–20 cells, Fig. 4B), our BLI detection technique was capable of detecting as few as 3 cancer cells in blood samples processed by RBC lysis. Indeed, the average radiance for the corresponding wells (3–20 cells) was significantly higher than the background of RBC-lysed whole blood (p = 0.02, Fig. 4A). These experiments demonstrate that highly sensitive BLI combined with RBC lysis is capable of detecting as low as 3 4T1-GL CTCs spiked in a mouse blood sample. Furthermore, using the linear correlation (Fig. S3B), we showed that this improved method can be used to quantify CTCs numbers in mouse blood, for CTC numbers higher than 3 cells per 100 µL blood sample.

### Monoclonality increases the detection sensitivity for low numbers of CTCs

To decrease the variability of the BLI signal for very low number of cancer cells spiked in blood samples, we hypothesized that a cell line of monoclonal origin would harbor a brighter and more homogeneous BLI signal than the FACs-sorted mixed population 4T1-GL. We selected the brightest single cell clone 4T1-F3 from a subpopulation of the 4T1-GL cell line (see Materials and Methods and Fig. 4C). The 4T1-F3 clonal population was on average 1.6 times brighter than the parental 4T1-GL population (Fig. 4C). When fewer than 23 4T1-F3 were spiked in 100-µL blood samples, our BLI detection method coupled with RBC lysis was capable of detecting as few as 2 cells per 100-µL blood sample. Indeed, the average radiance values for the corresponding wells (2–23 cells) were significantly higher (2.1–6.8 fold, p = 0.002) than the background of RBC-lysed whole blood. By contrast, the “unprocessed” 4T1-F3 spiked samples that were not lysed remained at the BLI intensity level of the background of unprocessed whole blood (Fig. 4D). Furthermore, we showed that the sensitivity of our CTC detection method was the same for cancer cells spiked in 1 mL of blood or in 100 uL of blood (Fig. S3A). This demonstrates our capability to detect low numbers of CTCs in larger blood volumes (1 mL) as well smaller blood volumes (100 µL) using our method. Using the linear correlation established between background-corrected BLI signals and number of CTCs, we showed that this improved method can be used to quantify as few as 2 CTCs in a 100-ul spiked mouse blood sample (Fig. S3C). The lower limits of reliable detection in an individual mouse blood sample was found to be 9 CTCs. Indeed, there was a sample with 9 spiked cancer cells that did not give a signal above blood background. All samples with ≥10 spiked cancer cells harbored BLI signals above background (n≥3 per data point).

**Figure 5:**
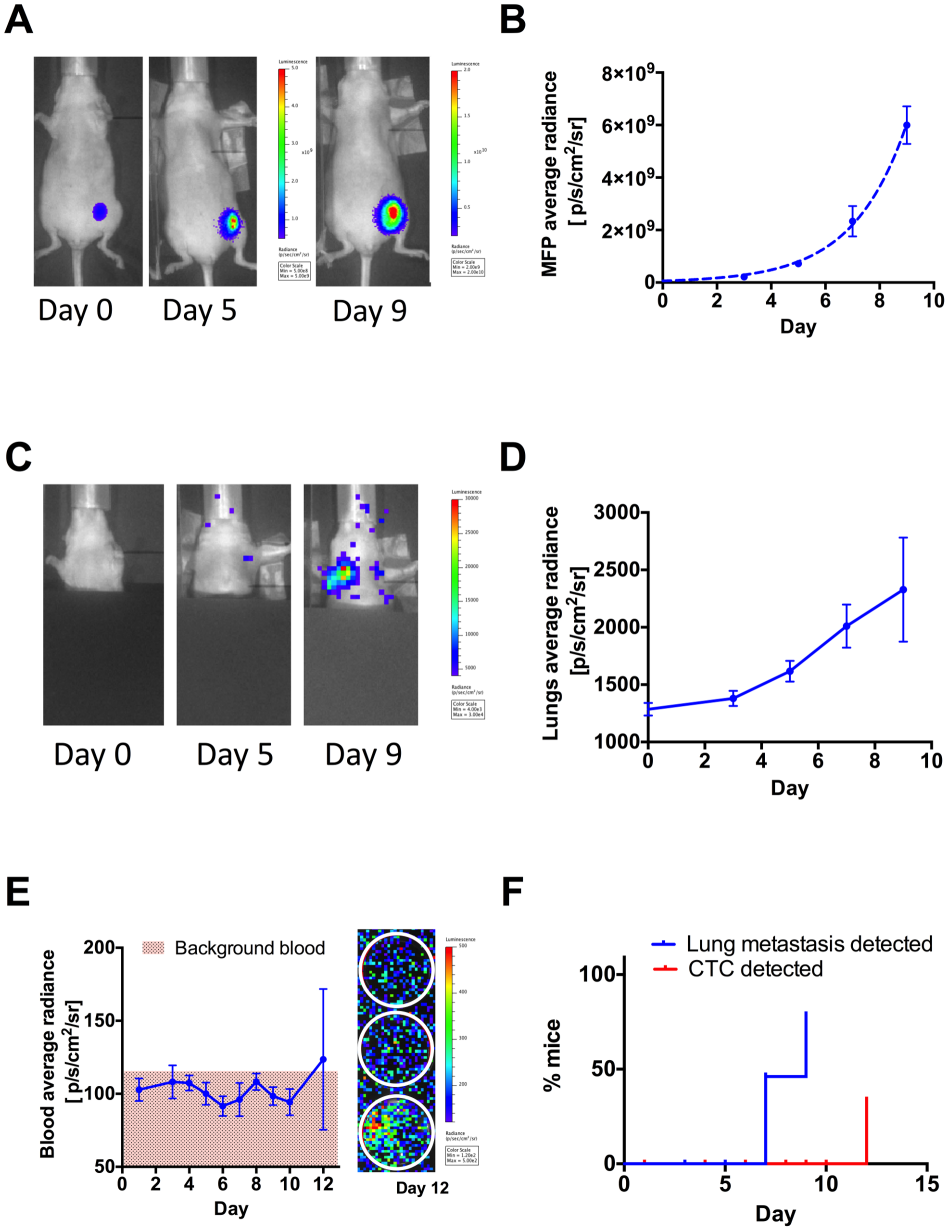
Bioluminescence imaging of primary tumor, metastatic growth and CTC dynamics in the 4T1-F3 orthotopic mammary tumor model. (**A**) Primary tumor growth in the mammary fat pad (m.f.p) as monitored using Bioluminescence (BLI) imaging, following implantation of 5×10^6^ 4T1-F3 cells in the fourth left mammary fat pad (image scales in p/s/cm^2^/sr), and (**B**) Corresponding quantification of BLI signal in the m.f.p area, shown as mean ± SEM and fit of the mean values to an exponential tumor growth equation (dashed line, R^2^ = 0.99). (**C**) Metastases in the upper body (lung area) as monitored using BLI in the same animals (image scales in p/s/cm^2^/sr) and (**D**) Corresponding quantification of the signal in the lung area, shown as mean ± SEM. (**E**) Corresponding quantification of blood BLI signal in 1-mL terminal blood samples from n = 3–4 animals/day (image scales in p/s/cm^2^/sr), shown as mean ± SEM. The image on the right shows BLI signal from CTCs in n = 3 terminal blood samples on day 12, (image scales in p/s/cm^2^/sr) (**I**) Statistical analysis showing the onset of lung metastasis and blood CTCs over time in the n = 40 animals.

We have shown that tumor cells are shed from the primary tumor between day 9 and day 12 in our 4T1-GL orthotopic tumor model (Fig. 2, Fig. 3). This time period is an ideal window to monitor CTCs in the blood on their way to giving rise to potential metastases. Since we were previously unable to detect CTCs in blood before the appearance of metastasis (Fig. 3F), we next sought to use our RBC lysis technique and 4T1-F3 metastasis model to focus on early tumor development and metastatic spread, up to day 12 post tumor inoculation. We orthotopically implanted 5×10^6^ 4T1-F3 cells in the murine m.f.p and monitored breast tumor growth by whole-body BLI over a period of 9 days in n = 40 mice. Primary tumor growth was exponential during this early tumor development phase (R^2^ = 0.75, Fig. 5A–B). We monitored the torso the animal for lung metastases that were observed as early as 7 days following tumor implantation (Fig. 5C–D) and increased up to 9 days for all mice (Fig. 5C). The first lung metastases could be detected at day 7 in 50% of the mice and at day 9 in ∼80% of animals (Fig. 5F). We collected 1-mL terminal blood samples from groups of n = 3–4 mice at daily intervals from day 1 to day 12. Those samples were processed by RBC lysis and imaged in our BLI imaging system as described previously. BLI signal was not detectable in terminal blood samples up to day 11. At the end of the study (day 12), we could detect the first CTCs present in blood samples in 33% of the mice (Fig. 5E–F). According to the background-corrected standard curve for 4T1-F3 CTCs (Fig. S3C), this signal is corresponding to 1.5 CTCs per mL blood (i.e., <3 CTCs in the approximate 2-ml mouse blood volume). Furthermore, CTCs only become detectable 3–5 days after the onset of metastasis. Finally, we note that there was stochasticity in the CTC seeding process, since CTCs were observed only in a fraction of the studied animals.

## Discussion

Mouse models of cancer metastasis, including the 4T1 mouse mammary carcinoma cell line, have been able to provide great insight into the metastatic process including invasion and intravasation [40], the evolution of primary tumor subpopulations [41] and the role of the immune system in metastatic spread [42]. Despite advances in our understanding of the metastatic process, the dynamics of CTCs over the timecourse of tumor progression, in particular before the appearance of metastasis, are not well characterized. To our knowledge, few preclinical assays allow for highly sensitive and specific repetitive assessment of live circulating tumor cells in mouse models of cancers, independently of specific surface markers.

In this study, we developed a method using bioluminescence imaging to detect and quantify low numbers of GL-expressing CTCs in mouse blood samples in a living mouse model of metastatic breast cancer. We were able to detect as few as 2 cancer cells in a mouse blood sample of up to 1 mL volume – roughly corresponding to 1 CTC per 2×10^6^ nucleated blood cells. Although the efficiency of cell recovery after RBC lysis may affect both the absolute value of our measurements and the lower limit of reliable detection, we have achieved a detection sensitivity comparable to that of the most sensitive CTC detection techniques, namely PCR-based and immunological assays (Cell Search, CTC-chip) which can detect 1 CTC in 10^6^–10^7^ nucleated blood cells [35], [43]. We note that the sensitivity of our method is significantly higher than flow-cytometry-based CTC detection methods, which can detect 1 CTC in 10^5^ nucleated blood cells [20]. In preclinical mouse studies, where CTC numbers are generally very low (<100 CTCs per mL of blood, [11]–[29]), being able to detect a single CTC in a blood sample is critical. A potential limitation of our BLI assay is the difficulty to determine the presence or absence of a single CTC using solely the IVIS system without confirmation using BLI microscopy.

As BLI signal can only be detected from live cells, our method has a clear advantage when compared to PCR-based and immunological assays that could potentially also assay dead cells present in blood. We demonstrated that live CTCs detected via our method could be isolated and identified by microscopy as single cells or clusters of CTCs (Fig. 3E). These live cells could potentially be cultured and expanded for further characterization [37], [38].

Furthermore, our BLI based CTC detection method is quick, requiring only the addition of the D-luciferin substrate after RBC lysis, and it is high-throughput, enabling simultaneous imaging of up to 96 100-µl blood samples. Most other CTC detection methods require each blood sample to be processed separately [35], [43]. Finally, because our BLI detection method does not rely on the expression of specific surface antigens or markers to detect the CTCs, we are capable of detecting all cancer cells in circulation that originated from the primary tumor and express the luciferase gene, and not just a subset of them.

Fluorescently labeled mouse models of metastasis allow for the same high degree of specificity as our BLI based method. In those models, CTCs originating from a primary tumor and expressing a fluorescent transgene such as GFP can be detected in a high-throughput manner using microfluidic devices, microscopy [44] or *in* *vivo* flow cytometers [27], [28], [45]. Detection of CTCs based on GFP fluorescence also allows for recovery of live CTCs at a relatively low cost, and unlike BLI, doesn’t rely on the addition of a substrate. Fluorescence imaging provides high sensitivity, in the nanomolar to picomolar range [30], allowing for the detection of single cells. However, BLI is 3–8 orders of magnitude more sensitive than fluorescence imaging [30]. This is mostly because fluorescence imaging can suffer from background signal due to autofluorescence, an important issue to consider when processing blood samples.

Several studies evaluating CTCs in mouse models of breast cancer were only able to detect CTCs after there was strong evidence of distant metastasis [20], [21]. When applying BLI to the daily detection of CTCs in the 4T1 orthotopic mammary carcinoma model, our findings demonstrate that early metastatic spread is correlated with the presence of CTCs in blood samples. This is in agreement with findings by Aslakson and Miller, who used clonogenic assays to weekly assess CTCs in terminal blood samples and lung metastasis in the 4T1 mouse model; they observed the presence of CTCs concurrently with lung metastases at day 7 post-tumor implantation [46]. Similarly, Schmidt et al. also observed an onset of CTCs in terminal cardiac punctures at the same time that metastasis appeared in the lungs, using flow cytometry approaches to count CTCs on a weekly basis in the blood of mice bearing another aggressive metastatic breast cancer cell line, 435/HAL [21]. A recent study using *in* *vivo* flow cytometry (IVFC) approaches evaluated CTC dynamics in mouse models of metastatic breast cancer and melanoma on a weekly basis [45]. However, in those studies, it is possible that their weekly assessment of CTCs missed the presence of CTCs at specific timepoints before the appearance of metastasis. Our study is the first to assess blood on a daily basis in a metastatic breast cancer model for the first 12 days before metastases were observed. Our findings suggest that there were no detectable CTCs in circulation before the appearance of BLI signal in the torso. Using experimental models of metastasis, in which cancer cells are injected in the tail vein and detected in the mouse ear, it has been shown that the average half-life of cancer cells in blood varies from 8 to 58 min [47]. Therefore, there is a short window of time to catch those cells using a blood draw. This has been confirmed by Juratli et al. who observed high variations in CTC numbers in consecutive IVFC 5-min imaging windows and in sequentially drawn patient blood samples [45]. We envision that this problem may be addressed in the future using continuous intravital imaging methods in freely moving small animals [34].

We observed a peak of CTC circulation at day 20, coinciding with increased growth of lung metastases and a decreased primary tumor growth (most likely due to necrosis). We showed that there was no significant linear correlation between blood BLI signal and lung metastasis signal (Fig. 3D). Since the lungs are located deeper behind the thoracic wall, our BLI imaging of the torso area might not accurately reflect lung metastasis. However, our results mirror those found by Schmidt et al. and Juratli et al. who also noted that CTC dynamics are not correlated with primary tumor growth dynamics or secondary lung metastasis growth [21], [45]. In our case, we were able to demonstrate a linear correlation between viable primary tumor size and CTC numbers independently of the stage of tumor growth (Fig. 3C). Furthermore, we observed patterns of 4T1-CTC dynamics over the timecourse of tumor metastasis that were very similar to those uncovered by other studies, harboring a peak in CTCs followed by a decrease in CTCs numbers in the bloodstream [21], [45], [46], [48]. Other studies have also observed two peaks of CTCs [45], [48] but this was not observed in our study. These interesting dynamics remain to be correlated with specific events in tumor development, as well as the capacity of those cells to survive in the circulation and extravasate.

Although BLI offers clear advantages for monitoring CTCs in animal models of metastasis, key limitations must still be addressed before we can address critical questions regarding circulation of cancer cells in the bloodstream. Firstly, our CTC BLI method is limited to animal models of cancer (in which cells must be transfected with the luciferase gene) and therefore cannot be directly translated to CTC detection in patient blood. Secondly, despite the gain in sensitivity after RBC lysis, we observed a slight decrease in correlation between BLI signal and spiked cell number (from R^2^ = 0.78 to R^2^ = 0.64, Fig. 4A) likely due to variable cell loss during the RBC lysis step. Therefore the RBC lysis step increased the sensitivity of our method but also increased its variability.

A third limitation is that, like all blood CTC assays, our limit of detection is limited by statistical considerations inherent to rare event detection, including (1) the number of samples collected at the same time from the same subject and (2) the limited volume of blood that can be sampled repetitively [49]. In our study, <10% of the total blood volume (TBV) of an animal can be collected every 1–2 weeks without negatively affecting the animal’s health. We have demonstrated here that CTCs are present at extremely low numbers (2–15 CTCs) in 100-µL blood samples (5% TBV) in 4T1-tumor bearing mice, even when the animals already presents metastasis. To gain more insight into pre-metastatic CTC dynamics, we were required to collect up to 1-ml terminal blood samples (50% TBV) in some studies, which precluded non-invasive longitudinal monitoring in the same animals. Based on a Poisson distribution of CTC [49] in a mouse with 40 CTCs in TBV (corresponds to 2 CTC/100-µL blood), we calculated that the probability of collecting at least 2 CTC in a single 100-µL blood sample is 59%. This relatively low probability, combined with other factors that we can’t account for, such as lysis of cells and cell death, may explain the very low numbers of CTCs detected in our study.

Our method allows the longitudinal assessment of CTC in animal models of cancer where many tumor parameters can be potentially be controlled and/or measured. When controlling for implanted primary tumor type and size, we identified a linear correlation between number of CTCs and viable primary tumor progression. Translated to the clinical setting, our result might suggest that such a relationship could exist in individual patients between primary tumor size and CTC numbers, highlighting the potential of CTC enumeration for predicting response to therapy. Promising clinical data is emerging correlating longitudinal assessment of CTCs in patient blood with tumor response to therapy in individual patients [50], [51]. More studies are needed to highlight the value of longitudinal CTC enumeration in cancer patients.

Our results based on the mouse 4T1-GL cell line might also extend to other breast cancer cell lines, including human cancer cell lines, with varying metastatic potential, as well as to other types of cancer. Applying our method to other models of cancers may highlight ideal characteristics of CTC detection assays for prognosis and help understand the role of CTCs dynamics in predicting tumor response to therapy. Understanding the appearance and dynamics of CTCs during the course of tumor development and treatment may help to supplement existing biomarker and imaging-based strategies to improve management of metastatic breast and other cancers.

## Supporting Information

Figure S1 **Fluorescence microscopy of a spiking experiment.** Fluorescence microscopy image of entire 1-µL drop of 4T1-GL labeled with CFSE, used for the purpose of counting the exact number of cancer cells spiked in a blood sample.
(TIFF)

Figure S2 **Quantification of BLI imaging for low numbers of cancer cells spiked in blood.** (**A**) Quantification of BLI imaging of low numbers (0–25) of 4T1-F3 cells spiked into 100 µL blood or 1 mL blood and processed by RBC lysis, shown as mean ± SEM (in 100 µL blood) or individual spiking experiments (in 1 mL blood). There was a significant linear correlation (R^2^ = 0.68 for 100 µL blood; R^2^ = 0.92 for 1 mL blood). (**B**) Quantification of the background corrected BLI signal for low number (0–50) of 4T1-GL cells spiked into 100 µL blood with or without RBC lysis. The line indicates a linear regression for unprocessed blood samples (R^2^ = 0.76) and RBC-lysed blood samples (R^2^ = 0.67). (**C**) Quantification of background corrected BLI signal of low numbers (0–25) of 4T1-F3 cells spiked into 100 µL blood followed by RBC lysis, shown as mean ± SEM. The line indicates a linear regression (R^2^ = 0.57) of the mean values and the red-dotted line shows the 95% confidence interval. The background corrected signals were calculated as follow: (Average radiance – Background average radiance)/(Background average radiance).
(TIFF)

Figure S3 **Dynamics of CTCs as measured by blood BLI.** (**A**)**–**(**G**) Dynamics of CTCs as measured by blood BLI (p/s/cm^2^/sr, red curve, right axis) and primary tumor growth as quantified by BLI (p/s/cm^2^/sr, blue curve, left axis) in individual 4T1-GL tumor bearing mice (n = 7). (**H**) Dynamics of CTCs as measured by blood BLI (p/s/cm^2^/sr, red curve, right axis) and primary tumor growth as quantified by BLI (p/s/cm^2^/sr, blue curve, left axis), shown as mean ± SEM.
(TIFF)
