# Supplementary figures and images for: Detection and Quantitation of Circulating Tumor Cell Dynamics by Bioluminescence Imaging in an Orthotopic Mammary Carcinoma Model
Source: PLoS One. 2014 Sep 4;9(9):e105079. doi: 10.1371/journal.pone.0105079 (PMC4154864; doi:10.1371/journal.pone.0105079)

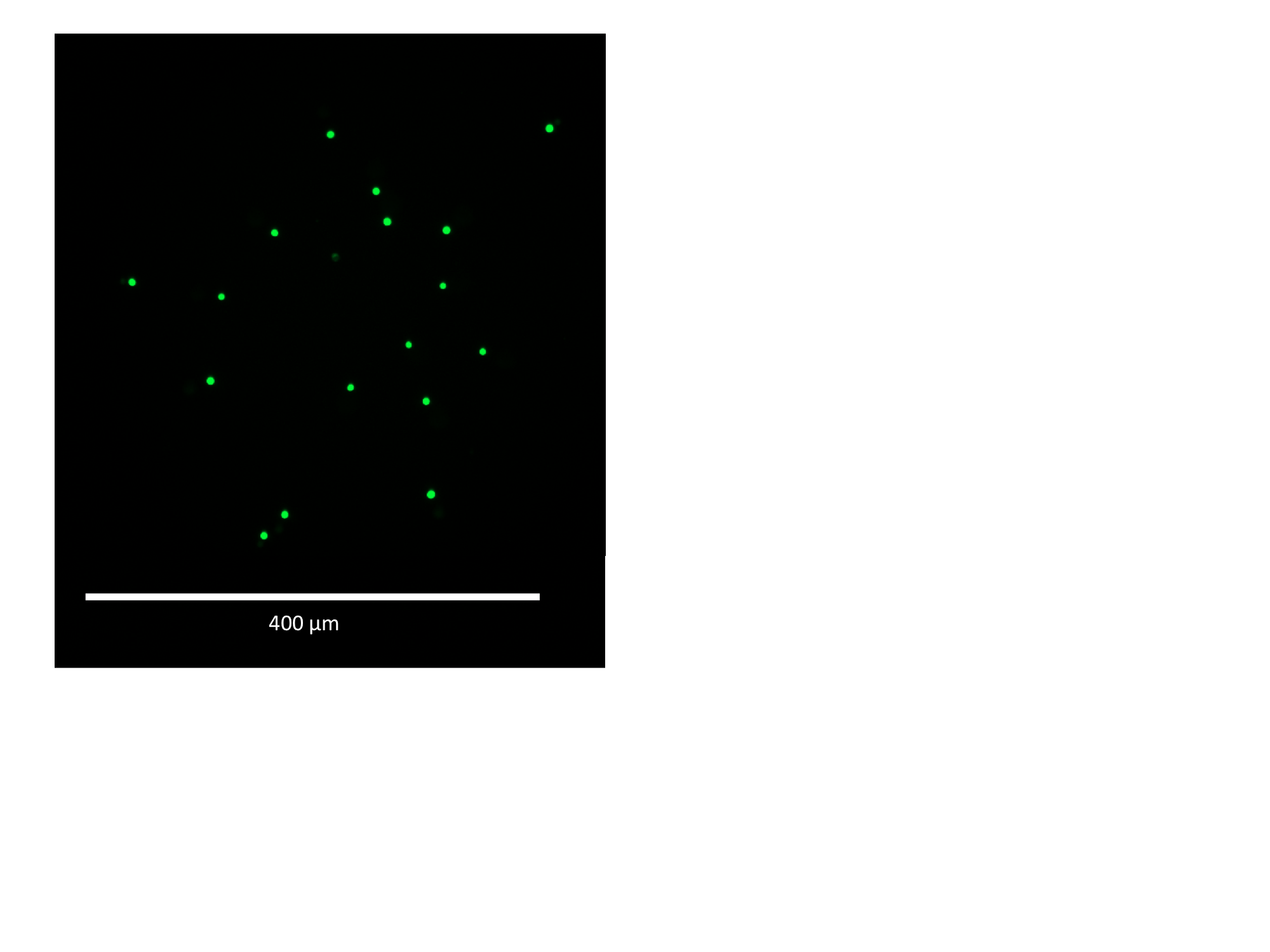

Supplement: Figure S1 — Fluorescence microscopy of a spiking experiment. Fluorescence microscopy image of entire 1-µL drop of 4T1-GL labeled with CFSE, used for the purpose of counting the exact number of cancer cells spiked in a blood sample. (TIFF) [file pone.0105079.s001.tiff]

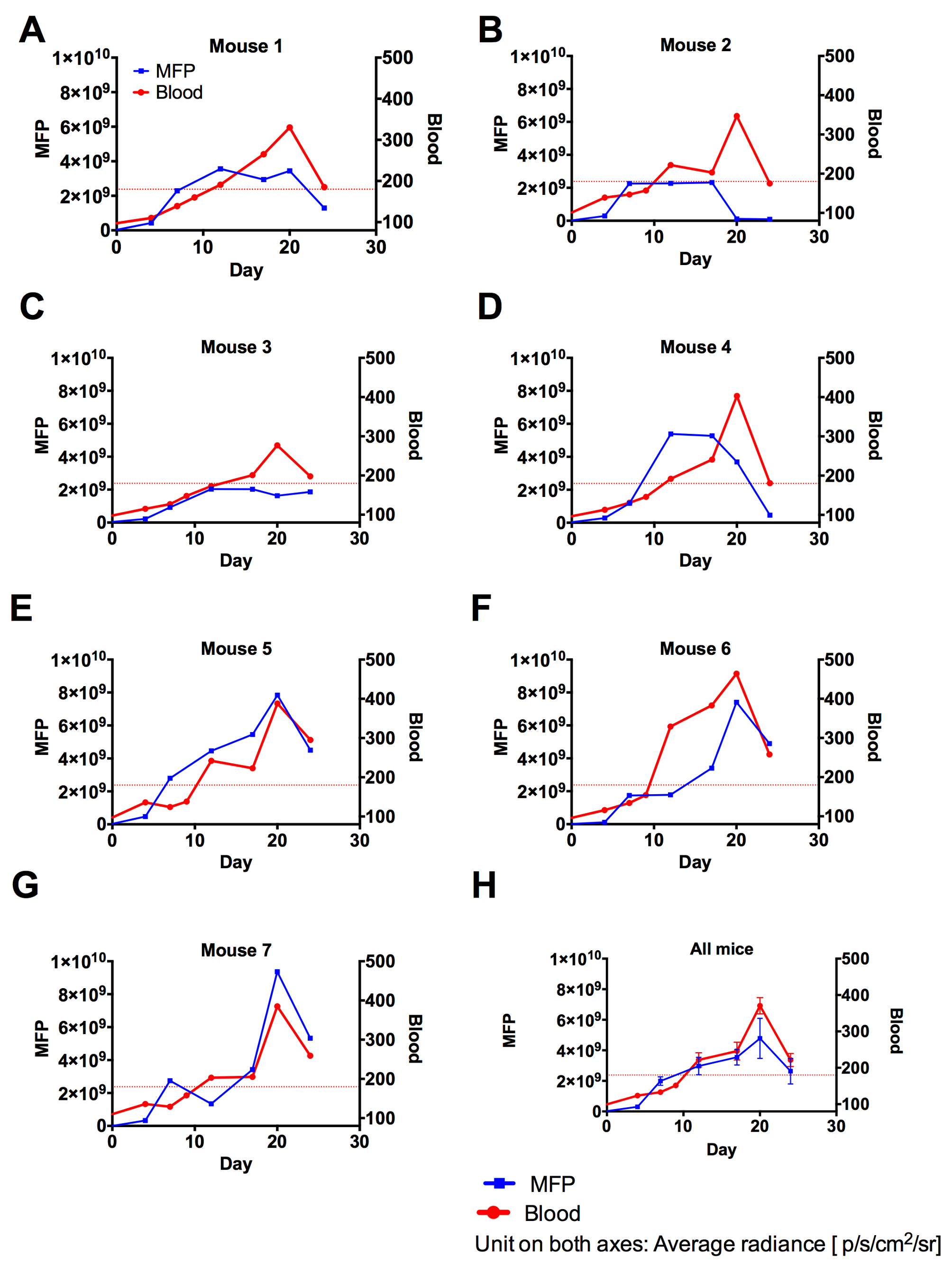

Supplement: Figure S2 — Quantification of BLI imaging for low numbers of cancer cells spiked in blood. (A) Quantification of BLI imaging of low numbers (0–25) of 4T1-F3 cells spiked into 100 µL blood or 1 mL blood and processed by RBC lysis, shown as mean ± SEM (in 100 µL blood) or individual spiking experiments (in 1 mL blood). There was a significant linear correlation (R2 = 0.68 for 100 µL blood; R2 = 0.92 for 1 mL blood). (B) Quantification of the background corrected BLI signal for low number (0–50) of 4T1-GL cells spiked into 100 µL blood with or without RBC lysis. The line indicates a linear regression for unprocessed blood samples (R2 = 0.76) and RBC-lysed blood samples (R2 = 0.67). (C) Quantification of background corrected BLI signal of low numbers (0–25) of 4T1-F3 cells spiked into 100 µL blood followed by RBC lysis, shown as mean ± SEM. The line indicates a linear regression (R2 = 0.57) of the mean values and the red-dotted line shows the 95% confidence interval. The background corrected signals were calculated as follow: (Average radiance – Background average radiance)/(Background average radiance). (TIFF) [file pone.0105079.s002.tiff]

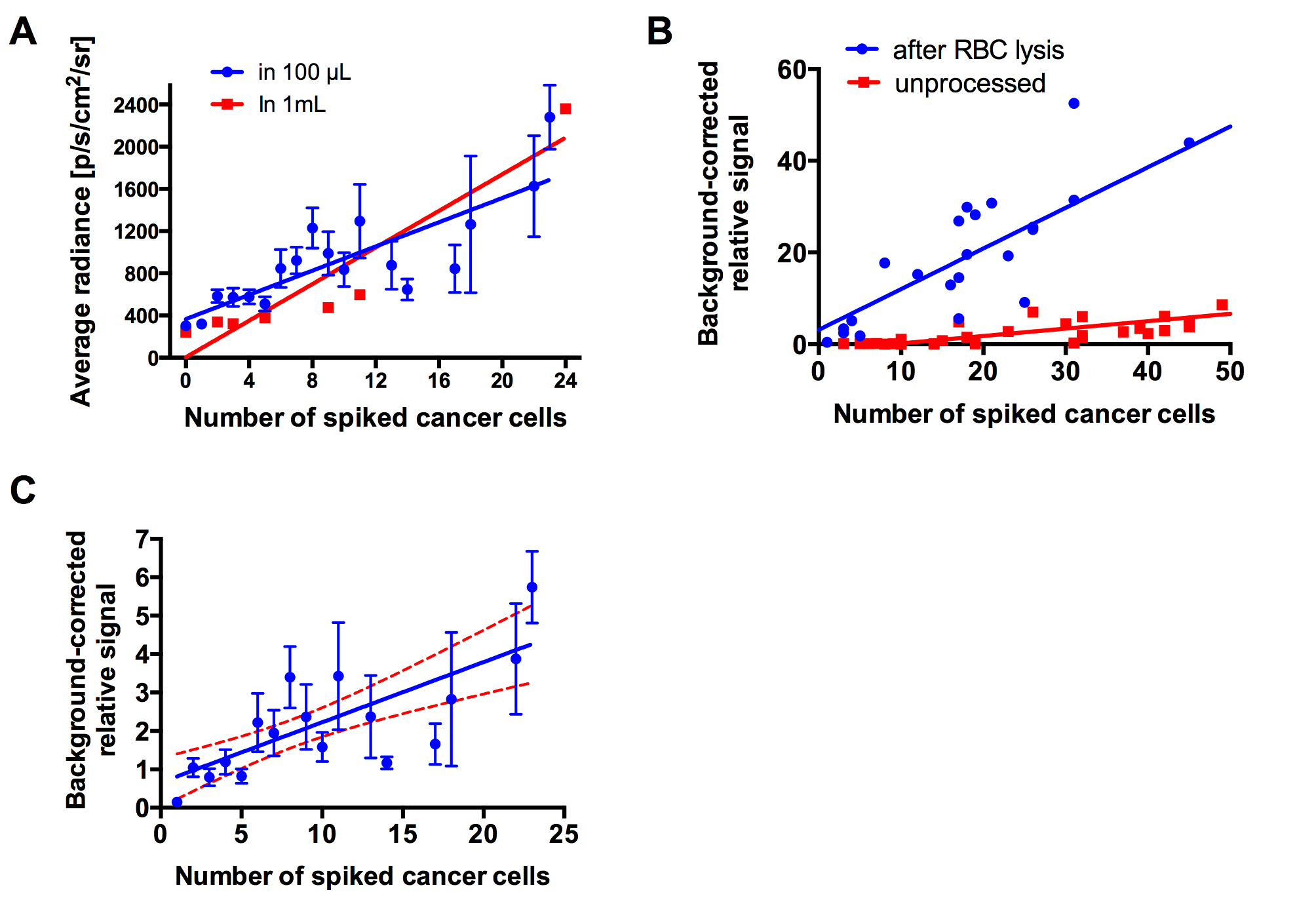

Supplement: Figure S3 — Dynamics of CTCs as measured by blood BLI. (A)–(G) Dynamics of CTCs as measured by blood BLI (p/s/cm2/sr, red curve, right axis) and primary tumor growth as quantified by BLI (p/s/cm2/sr, blue curve, left axis) in individual 4T1-GL tumor bearing mice (n = 7). (H) Dynamics of CTCs as measured by blood BLI (p/s/cm2/sr, red curve, right axis) and primary tumor growth as quantified by BLI (p/s/cm2/sr, blue curve, left axis), shown as mean ± SEM. (TIFF) [file pone.0105079.s003.tiff]
